# Mass spectrometric characterization of cyclic dinucleotides (CDNs) in vivo

**DOI:** 10.1007/s00216-021-03628-6

**Published:** 2021-09-02

**Authors:** Andrea Annibal, Roberto Ripa, Eugen Ballhysa, Christian Latza, Nadine Hochhard, Adam Antebi

**Affiliations:** 1 Max Planck Institute for Biology of Ageing, Cologne, Germany; 2 Cologne Excellence Cluster on Cellular Stress Responses in Aging-Associated Diseases (CECAD), University of Cologne, Cologne, Germany

**Keywords:** 2′3′ cGAMP, *Nothobranchius furzeri*, Mass spectrometry, cGAS

## Abstract

Cyclic dinucleotides (CDNs) are key secondary messenger molecules produced by cyclic dinucleotide synthases that trigger various cellular signaling cascades from bacteria to vertebrates. In mammals, cyclic GMP-AMP synthase (cGAS) has been shown to bind to intracellular DNA and catalyze the production of the dinucleotide 2′3′ cGAMP, which signals downstream effectors to regulate immune function, interferon signaling, and the antiviral response. Despite the importance of CDNs, sensitive and accurate methods to measure their levels in vivo are lacking. Here, we report a novel LC-MS/MS method to quantify CDNs in vivo. We characterized the mass spectrometric behavior of four different biologically relevant CDNs (c-di-AMP, c-di-GMP, 3′3′ cGAMP, 2′3′ cGAMP) and provided a means of visually representing fragmentation resulting from collision-induced dissociation at different energies using collision energy breakdown graphs. We then validated the method and quantified CDNs in two in vivo systems, the bacteria *Escherichia coli* OP50 and the killifish *Nothobranchius furzeri*. We found that optimization of LC-MS/MS parameters is crucial to sensitivity and accuracy. These technical advances should help illuminate physiological and pathological roles of these CDNs in in vivo settings.

**Supplementary Information:**

The online version contains supplementary material available at 10.1007/s00216-021-03628-6.

**Figure:**
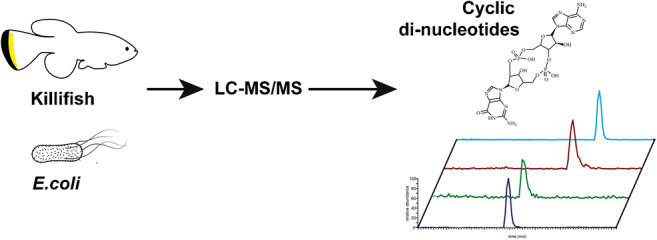
Graphical abstract

## Introduction

Cyclic dinucleotides (CDNs) are a class of molecules that serve as important secondary messengers conserved across taxa. Bacterial CDNs form through the cyclization of two nucleotides via a phosphodiester bond that links the C3′ of one pentose ring with the C5′ of the other nucleotide, resulting in a 3′5′ cyclic dinucleotide. Dinucleotide cyclase vibrio (DncV)–like nucleotidyl transferases (CDNTases) catalyze such reactions in prokaryotes and are best known for the synthesis of purine cyclic-bis dimeric GMP and c-di-AMP, heterocyclic 3′3′ cGMP-AMP (cGAMP), but have been recently shown to generate pyrimidine derivatives as well [1]. These molecules regulate processes as diverse as potassium ion channel expression, osmoprotection, cell-wall homeostasis, DNA repair, and antibiotic resistance [2, 3]. Most notably, c-di-GMP was identified as playing a central role in biofilm production, whereas 3′3′ cGAMP modulates bacterial chemotaxis and protects against viral infection [1, 4–6].

Vertebrate cyclic GMP-AMP synthase (cGAS) is best known for its role in nucleic acid signaling. In particular, it senses both foreign and cytosolically mislocalized self-double-stranded (ds)DNA and triggers a defense response [7, 8]. Upon binding dsDNA, cGAS generates a unique 2′3′ cGAMP isomer, which activates the endoplasmic reticulum resident receptor Stimulator of Interferon Genes (STING) [9]. STING itself responds to either endogenous or bacterially produced CDNs, and translocates to the Golgi, where it interacts with TBK1 kinase to induce IRF3-dependent type I interferon and NF-κB transcriptional responses, thereby mounting antiviral immunity and antimicrobial defense [10]. Recent work also suggests that cGAS/STING signaling is involved in sensing retroviruses, including HIV-1 and HIV-2, as well as the RNA virus COVID-19 [11, 12]. Further, aberrant activation of the pathway, triggered by the presence of intracellular chromatin micronuclei or mitochondrial DNA, is also related to chronic inflammation, cancer, and cellular senescence [13–15]. Thus, the cGAS/Sting pathway has emerged as central to nucleic acid dynamics, immunity, and aging.

Despite significant advances in elucidating the physiology of the cGAS/STING pathway, methods to monitor CDNs’ dynamics in living organisms remain limited. Most studies rely on comparative measurement of downstream components, such as type I interferons (IFN-I) and IFN stimulated genes (ISGs) as indirect indicators of cGAS activity. Measurement of the CDNs themselves has been quantified by competitive ELISA [9], monoclonal antibodies, thin-layer chromatography, radioisotope, ion-exchange chromatography [16–19], and an RNA-based fluorescent biosensor [20]. However, most of these methods fall short on accuracy, sensitivity, or specificity.

Liquid chromatography-mass spectrometry (LC-MS) is another method used to measure endogenous cell or tissue cGAMP levels. Work of Lorenzetti et al. and Pajio et al. described a LC-MS-based methodology for the detection of these species in biological samples [21, 22]. Nonetheless, these LC-MS studies lack a structural mass spectrometric characterization of the CDNs, which is the key to develop more sensitive and accurate methods for their quantification.

In this work, we initiated a mass spectrometry–based investigation of four CDNs and optimized an LC-MS/MS method for their quantification. We performed a quantitative comparison of ion intensities in tandem mass spectra and analysis of energy-dependent (or energy-resolved) curves for structure analysis. These optimized parameters were used to develop a new MRM method, which was validated by quantifying CDNs in the bacteria *Escherichia coli* OP50. Moreover, we quantified 2′3′ cGAMP in tissues of the killifish *Nothobranchius furzeri*, an important emerging vertebrate model organism for studying aging and disease. These advances should greatly facilitate the accurate and sensitive quantification of cyclic dinucleotides in in vivo settings.

## Material and methods

### Chemicals

UHPLC-grade water, formic acid, and UHPLC-grade acetonitrile were purchased from Biosolve Valkenswaard, Netherlands. Chloroform was purchased from Merck KGaA (Darmstadt, Germany). UHPLC-grade methanol and 2′3′ cGAMP sodium salt (SML1229)**,** 3′3′ cGAMP sodium salt (SML1232), c-di-AMP sodium salt (SML1231), c-di-GMP sodium salt (SML1228), adenosine-^13^C_10_,^15^N_5_-5′-monophosphate lithium salt (650676), Tris hydrochloride, NaOH, KCl, ethylenediaminetetraacetic acid (EDTA), and tricaine methanesulfonate were purchased from Sigma-Aldrich, GmbH.

### Bacteria culture

*E. coli* OP50 bacteria were grown overnight (18 h) in LB media composed of 10 g/L Bacto tryptone (Sigma 95,039), 5 g/L Bacto yeast extract (BD 212720), and 5 g/L NaCl. Bacteria were centrifuged for 30 min at 4000 relative centrifugal force (rcf) at 4 °C. Pellets were frozen in liquid nitrogen and stored at – 80 °C.

### Killifish husbandry and tissue extraction

All experiments were performed using the killifish strain GRZ-AD [23]. All fish were individually housed in single 2.8-L tanks connected to a water recirculation system receiving 12 h of light and 12 h of darkness every day. Temperature was maintained at 27.5 °C. Adult killifish were fed twice a day with 1.1 mm pellet (BioMar) and brine shrimps (*Artemia*). For tissue collection, fish were euthanized with an overdose of tricaine methanesulfonate (TMS, MS-222) (0.4 mg/mL). Liver and gut tissues were immediately extracted by dissection, frozen in liquid nitrogen, and stored at – 80 °C.

### Fish transgenesis and genotyping

To generate the cGAS^−/−^ fish line, we used CRISPR/Cas9 genome engineering, targeting the conserved cGAS DNA-binding region with guide RNAs using the algorithm CHOPCHOP (https://chopchop.cbu.uib.no/). One-cell-stage killifish embryos were injected with ~ 2–3 nL of a solution containing 200 ng/μL of Cas9 protein (IDT: 1081060), 20 ng/μL of sgRNA (IDT: Alt-R® CRISPR-Cas9 sgRNA, target specific sequence: GCATTGAAACGTGATCCAAC), 200 mM KCl, and 10% (v/v) red-phenol. Juvenile fish (~ 3 weeks old) were genotyped by fin clipping. The tissue samples were first digested for 1 h at 98 °C in alkaline buffer (NaOH 25 mM, EDTA 0.2 mM) under constant shaking, and then 1 volume of Tris-HCl buffer (pH 5.5) was added to rebalance the pH. One microliter of solution was used as a template for a standard 25-μL PCR (DreamTaq Green PCR Master Mix, ThermoFisher) using the forward (Fw: GTTAAGGAACCCCTTCGCACT) and reverse (Rw: TTGCCGTCATCTCCCATTCTG) primers corresponding to the cGAS gene. The PCR products (554 bp) were purified from an agarose gel and sequenced (Supplementary Figs. 1, 2).

### Cyclic dinucleotide extraction

**Scheme 1:**
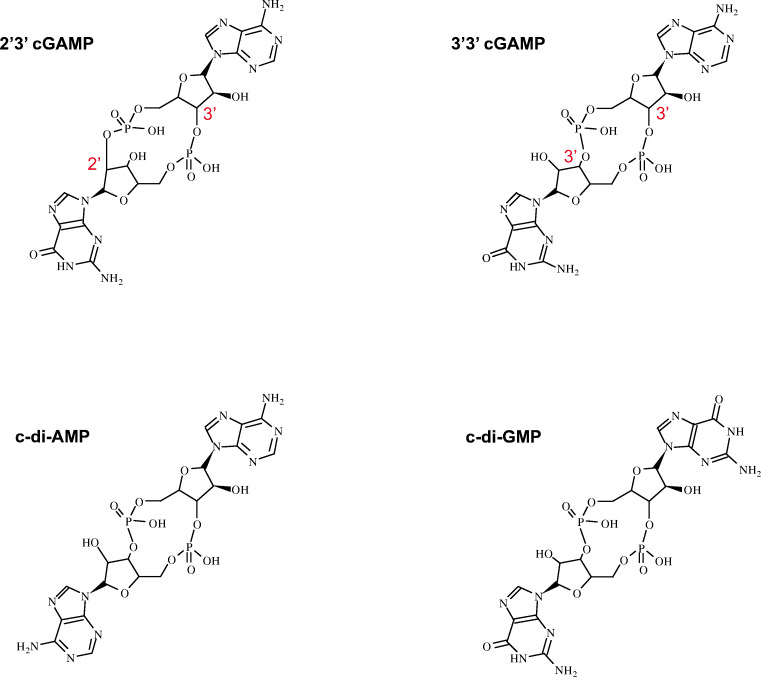
Molecular structure of four main cyclic dinucleotides (CDNs) produced in bacteria and vertebrates

CDNs were extracted from bacteria and from killifish by modification of our previous protocols [24, 25] (Scheme 1). Frozen tissues and bacteria pellets underwent up to three freeze/thaw cycles in liquid nitrogen and were then homogenized by bead beatings for 20 min at 50 oscillations/s at 4 °C using the Qiagen tissue lyser. Protein concentration was measured with the BCA kit (23225, ThermoFisher). Prior to extraction, 5 ng of an internal standard (adenosine-^13^C_10_,^15^N_5_–5′-monophosphate) was added. A volume corresponding to 300 μg of protein was subjected to Bligh and Dyer extraction by adding 100 μL chloroform/methanol 2:1 *v*/v. Samples were rotated for 1 h at 4 °C and centrifuged for 10 min at 10.000 rpm. The supernatant was transferred to a new tube and dried under a Speedvac. Samples were resuspended in 20 μL of 1% v/v acetonitrile in water with 0.1% v/v formic acid. Five microliters of the sample was injected into the LC-MS/MS system.

### LC-MS/MS instrumentation and conditions

Identification and relative quantification of cyclic dinucleotides were performed on a triple quadrupole mass spectrometer (QqQMS) (TSQ Altis, ThermoFisher Scientific GmbH, Bremen, Germany). Standards were dissolved in water and separated by reverse-phase column (XSelect HSS T3 2.5 μm 2.1 × 100 mm, Waters) using a binary pump system (Vanquish, ThermoFisher Scientific GmbH, Bremen, Germany) with solvent A as water with 0.1% v/v formic acid and eluent B as acetonitrile with 0.1% v/v formic acid, using a gradient modified from previous work [21].

Briefly, the gradient began with 1% eluent B and ramped up to 5% in 30 s. The gradient increased up to 50% eluent B in 5 min and reached 60% eluent B in 1 additional minute. At 7 min, it reached 90% eluent B and was held for 2 min. The gradient then decreased to 20% eluent B in 5 min and was held for 2 min at 1% eluent B. The total time was 16 min. The flow rate was 0.2 mL/min. The column temperature was set at 30 °C and the auto sampler temperature was set to 4 °C. Data was analyzed using Xcalibur version 4.0 and Trace Finder version 4.1.

### ESI-MS/MS conditions

ESI ionization parameters were as follows: 3.5 kV, 25 a.u. sheath gas, 5 a.u. auxiliary, and 350 °C transfer ion capillary. All the spectra were acquired in positive ion mode.

Full scans were acquired from 150 to 900 *m*/*z* with a scan rate of 1000 Da/s using a resolution of Q1 of 0.7 *m*/*z*. The product ion scans were obtained for each individual precursor using a scan rate of 1000 Da/s, using Q1 resolution of 0.7 *m*/*z* and Q3 resolution of 0.7 *m*/*z*. Tandem mass spectra were obtained by CID, using argon set at 1.5 mTorr with collision energy between 0 and 50 V. Multiple reaction monitoring (MRM) measurements using ESI-MS/MS were achieved with the following parameters: Q1 resolution 0.7 *m*/*z* and Q3 resolution 1.2 *m*/*z*. Different collision energies were used for each compound as indicated in the main text. Unique combinations of Q1 and Q3 *m*/*z* values were used to quantify cyclic dinucleotides (see main text). The relative response for each cyclic dinucleotide was calculated by dividing the peak area of the analyte to the internal standard peak area (adenosine-^13^C_10_,^15^N_5_-5′-monophosphate, *m*/*z* 407.08) and further normalized to protein concentration. The internal standard was used to compensate for variability in signal intensity due to ion suppression caused by matrix components that may influence the efficiency of ionization.

### Statistical analysis

GraphPad Prism Version 7.0c software was used for graphics and statistical testing.

## Results

**Fig. 1:**
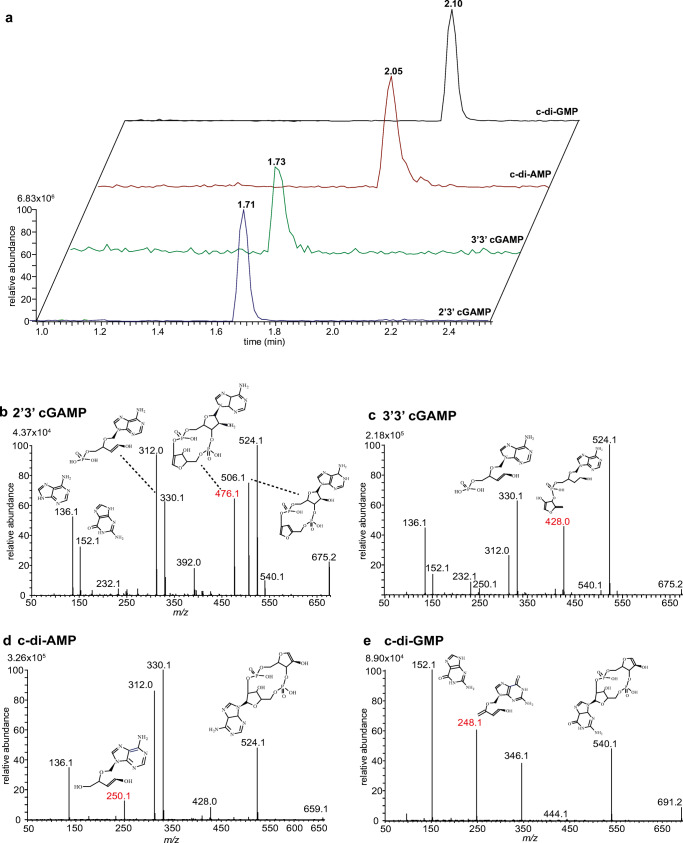
Characterization of CDNs by LC-MS/MS. **a** Extracted ion chromatograms (EIC) of CDN standards at concentration 100 nmol/L analyzed by LC-MS (full scan). Tandem mass spectra of 2′3′ cGAMP (**b**), 3′3′ cGAMP (**c**), c-di AMP (**d**), and c-di-GMP (**e**), using 30 V CE. Fragments used for the LC-MS/MS quantification are highlighted in red. The proposed fragments are designated as neutral molecules

We employed UHPLC coupled with ESI-MS/MS for the investigation of cyclic dinucleotides in vivo (Scheme 1). We first characterized the resolution of authentic standards of CDN molecular species via reversed-phase liquid chromatography using full scan acquisition (Fig. 1). Single protonated ions [M + H]^+^ were chosen for the analysis. Both heterocyclic isomers, 2′3′ cGAMP and 3′3′ cGAMP, displayed mono protonated ions at *m*/*z* 675.1 with a retention time of 1.71 min and of 1.73 min, respectively (Fig. 1a). Cyclic-di-AMP and c-di-GMP showed single protonated ions at *m*/*z* 659.1 and 691.2 and eluted at 2.05 min for c-di-AMP and 2.10 min for c-di-GMP. Despite their co-elution, it was still possible to distinguish c-di-AMP from c-di-GMP due to their different *m*/*z* values. Nonetheless, in order to discriminate between 2′3′ and 3′3′ cGAMP, we performed product ion scan experiments to retrieve unique fragments for each compound.

Product ion scans of the four different CDNs were initially performed using the same collision energy (30 V) (Fig. 1b–e). The tandem mass spectra obtained by collision-induced dissociation (CID) of 2′3′ cGAMP exhibited several dominant daughter ions (Fig. 1a). The ion at *m*/*z* 540.1 was assigned to C_15_H_20_N_5_O_13_P_2_^+^, whereas the ion at *m*/*z* 524.1 corresponded to C_15_H_20_N_5_O_12_P_2,_^+^ and, by subsequent water loss, led to the formation of the ion at *m*/*z* 506.1. The ion at *m*/*z* 476.1 corresponded to C_14_H_16_N_5_O_10_P_2_^+^, and the ion at *m*/*z* 330.1 was assigned to C_10_H_13_N_5_O_6_P^+^. Water loss from the latter ion led to a product ion at *m*/*z* 312.0. The tandem mass spectra of C_10_H_13_N_5_O_6_P^+^ resulted in the appearance of ion at *m*/*z* 152.1 (C_5_H_6_N_5_O^+^) and *m*/*z* 136.1 (C_5_H_6_N_5_^+^), which belong respectively to the nucleobases, guanine and adenine.

The product ion spectra of 3′3′ cGAMP were similar to that of 2′3′ cGAMP (Fig. 1c). The ion at *m*/*z* 524.1 (C_15_H_20_N_5_O_12_P_2_^+^) was also present in the spectra as well as the ion pair at *m*/*z* 330.1 and 312.0. These product ion spectra also contained unique fragments, such as the ion at *m*/*z* 428.0 (C_15_H_19_N_5_O_8_P^+^), which was absent in the 2′3′ cGAMP tandem mass spectra. These data indicate that each of the two isomers has specific marker ions that can be used to distinguish the two molecular species.

The tandem mass spectra of c-di-AMP were dominated by the presence of the ion at *m*/*z* 524.1 (C_15_H_20_N_5_O_12_P_2_^+^), and the ions at *m*/*z* 330.1 and *m*/*z* 312.0 (Fig. 1d). The product ion at *m*/*z* 250.10 was assigned to C_10_H_12_N_5_O_3_^+^. As expected, the tandem mass spectra displayed only a single ion at *m*/*z* 136.1 as both of the nucleotide moieties are adenosine.

The product ion scan of c-di-GMP revealed fewer fragments in comparison to other CDNs (Fig. 1e). We could identify the product ion at *m*/*z* 540.1 and the ion at *m*/*z* 346.1. This CID spectra displayed a unique fragment for c-di-GMP, namely at *m*/*z* 248.1, which corresponded to C_10_H_10_N_5_O_3_^+^. The ion at *m*/*z* 152.1 was present only in this tandem mass spectra, and assigned to guanine.

**Fig. 2:**
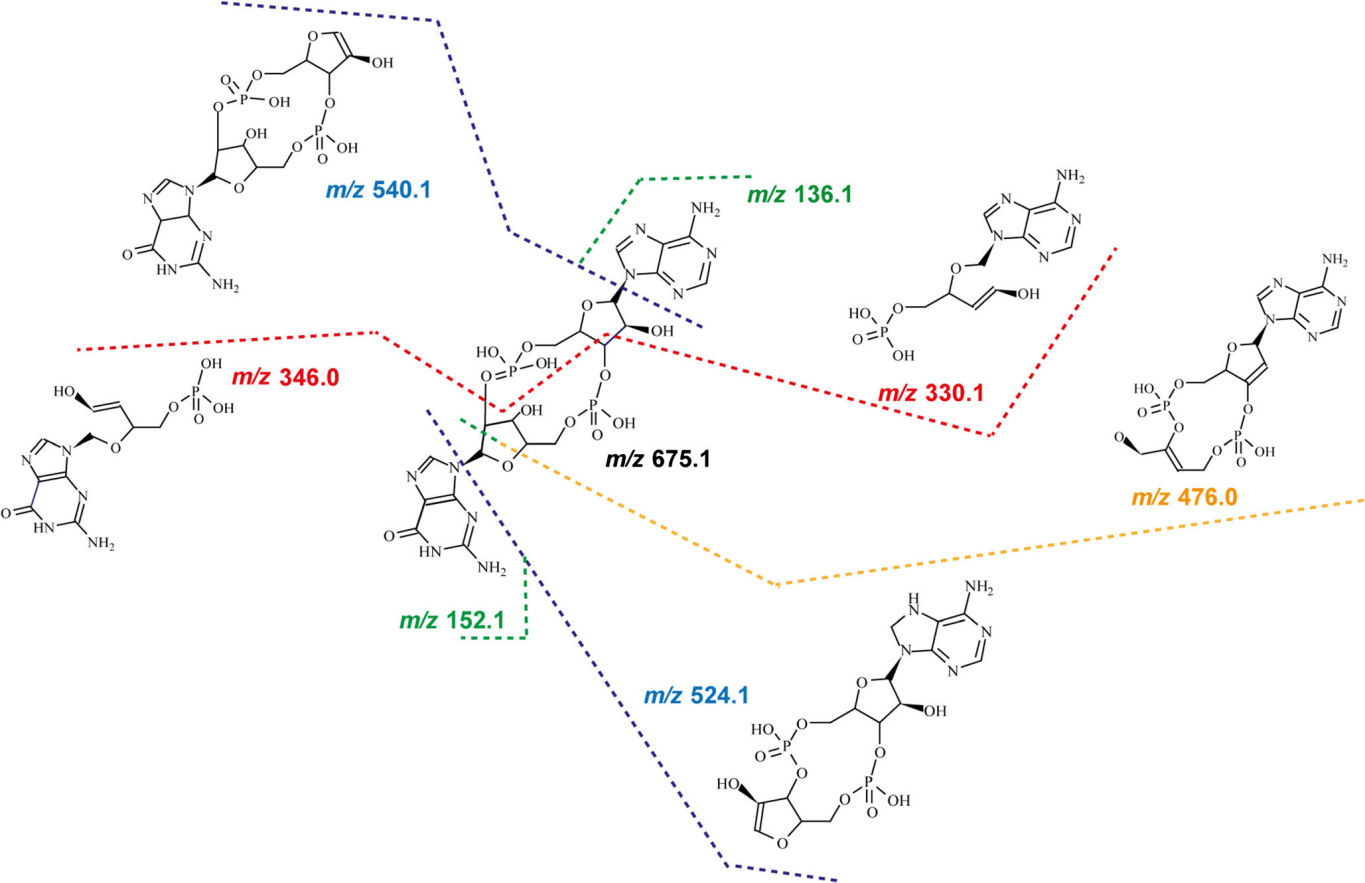
Fragmentation pattern of CDNs. Several preferential bond breakages occur, including cleavage between the sugar moiety and the nucleobase (green and blue), at the sugar ring (orange), and at the corresponding C3′ of the pentose ring with the C5′ of the phosphate group (red). The proposed fragments are designated as neutral molecules

In sum, the analysis of the four product scans allowed us to identify specific fragmentation patterns of CDNs upon CID (Fig. 2), with three main sites for breakage. The first cleavage occurs at the bond between the nucleobase and the ribose moiety. By loss of the nucleobase, two ion pairs are formed, 152.1/524.1 by loss of guanine and 136.1/540.1 by loss of adenine. The second ion pair is generated by cleavage at two bonds: between the C2′ of the pentose ring with the C5′ bond of the other nucleotide, and between the C3′ ribose ring and the C5′ of the second nucleotide. This breakage leads to ring opening, giving rise to the ions at *m*/*z* 330.1 and at *m*/*z* 346.0.

Remarkably, we observed breakage of a third bond, which occurred only at the sugar moiety of guanosine, leading to the formation of the fragment ion at *m*/*z* 476.0. In none of the MS/MS spectra were we able to detect the counterpart ion for breakage of the adenosine sugar moiety (expected ion at *m*/*z* 492.1).

**Fig. 3:**
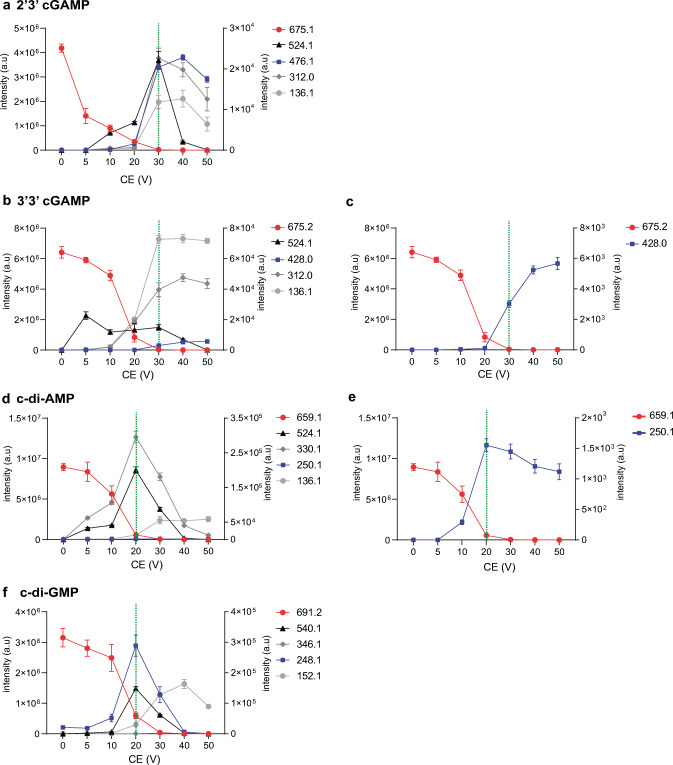
Breakdown curves of selected product ions for CDNs. Precursor and product ion yields are plotted as a function of collision energy (CE) settings for 2′3′ cGAMP (**a**), 3′3′ cGAMP (**b**), with zoom in on specific ion products for 3′3′ cGAMP (**c**), c-di-AMP (**d**), with zoom in on specific ion product for c-di-AMP (**e**), and c-di-GMP (**f**). Precursor ion intensity is plotted on the left *y*-axis, and product ion intensities are plotted on the right *y*-axis. Collision energies selected for the MRM method are indicated by the dotted green bar

Examining various energy-resolved curves provides a simple and valuable approach to select the best transition for multiple reaction monitoring (MRM). To optimize detection, we therefore examined the collision energy (CE)–breakdown curves (or energy resolved) of the four CDNs, retrieving fragment ions that might differentiate the isomers (Fig. 3). We plotted the intensity of the fragment ions upon varying the CE over a range of 0–50 V.

The breakdown curves for 2′3′ cGAMP revealed that most of the product ions reached a peak at CE of 30 V, which then decreased at higher voltages (Fig. 3a). As expected, the intensity of the precursor ion inversely decreased, reaching zero intensity at CE of 30 V.

The breakdown curves for 3′3′ cGAMP exhibited a different CE profile. The intensity of the fragment ions *m*/*z* 136.1 and 312.0 increased at CE of 30 V, but increased even further at higher voltages of 40 and 50 V (Fig. 3b). The intensity of the ion at *m*/*z* 428.0 also increased at higher voltages (Fig. 3c). Thus, 2′3′ cGAMP and 3′3′ cGAMP positional isomers can be additionally differentiated by their CE-breakdown curves.

CE-breakdown curves for c-di-AMP and c-di-GMP revealed different profiles compared to the heterocyclic molecules. In both curves, the daughter ions reached a peak at CE of 20 V. The CE curve of c-di-AMP showed a slower decrease for the ions 330.1 and 136.1, from CE at 20 V to CE at 40 V (Fig. 3d). The ion at *m*/*z* 250.1 had a maximum intensity at CE of 20 V and slowly decreased at higher CE (Fig. 3e).

The CE curve profile of c-di-GMP for the fragment ion at *m*/*z* 248.1 increased at CE of 10 V with a maximum at CE of 20 V, then rapidly decreased at higher voltage (Fig. 3d). The ions at m/z 540.1 and 152.1 also increased at CE of 20–30 V and decreased at CE of 40 V.

Based on the CE-breakdown curves, we selected the optimal collision energy at the maximum intensity of the fragment ion curve and the minimum of the precursor ion curve. Hence, we used a collision energy of 30 V for 2′3′ cGAMP and 3′3′ cGAMP, and 20 V for c-diAMP and c-di-GMP. Furthermore, unique fragments were chosen for each compound. We selected fragment ion at *m*/*z* 476.01 for 2′3′ cGAMP, ion at *m*/*z* 428.0 for 3′3′ cGAMP, ion at *m*/*z* 250.1 for c-di-AMP, and ion at *m*/*z* 248.1 for c-di-GMP. Thus, using the above optimized parameters, we created a new MRM-based method (LC-MS/MS) to distinguish these moieties.

Limits of detection (LOD) and quantification (LOQ) were determined from serial dilutions of the seven standards ranging from 10 pg/mL to 10 ng/mL (Tables 1 and 2). All external calibration curves exhibited high levels of linearity (> 0.90). Further, this method resulted in high intraday (5–9%) and interday precision (8–12%) for LC-MS/MS. Standards were also spiked into killifish samples prior to liquid-liquid extraction to calculate the recovery rate, which exceeded 83%.

**Table 1:** Intraday interday error and recovery of cyclic dinucleotides

	Intraday *n* = 5			Interday *n* = 5			
Compound name	Average area	STD	%RSD	Average area	STD	%RSD	Recovery
2′3′ cGAMP	4.45E+04	2349.936	5.28	4.79E+03	595.3594	12.41	91%
3′3′ cGAMP	5.32E+04	5207.004	9.79	6.45E+03	831.8047	12.90	83%
c-di-AMP	5.10E+04	4676.97	9.16	8.07E+03	764.1128	9.47	89%
c-di-GMP	2.95E+04	1659.272	5.61	4.79E+03	420.0636	8.77	85%

**Table 2:** Limits of detection, quantification, and calibration slopes of cyclic dinucleotides

Compound name	LOQ (ng/mL)	LOD (ng/mL)	Calibration slopes	
2′3′ cGAMP	0.17	0.46	*y* = 156.95*x* + 285.34	*R*^2^ = 0.94
3′3′ cGAMP	0.22	0.60	*y* = 763.49*x* + 592.25	*R*^2^ = 0.95
c-di-AMP	0.26	0.69	*y* = 1390.5*x* − 21,342	*R*^2^ = 0.95
c-di-GMP	0.19	0.50	*y* = 1157.1*x* − 6467.4	*R*^2^ = 0.97

**Fig. 4:**
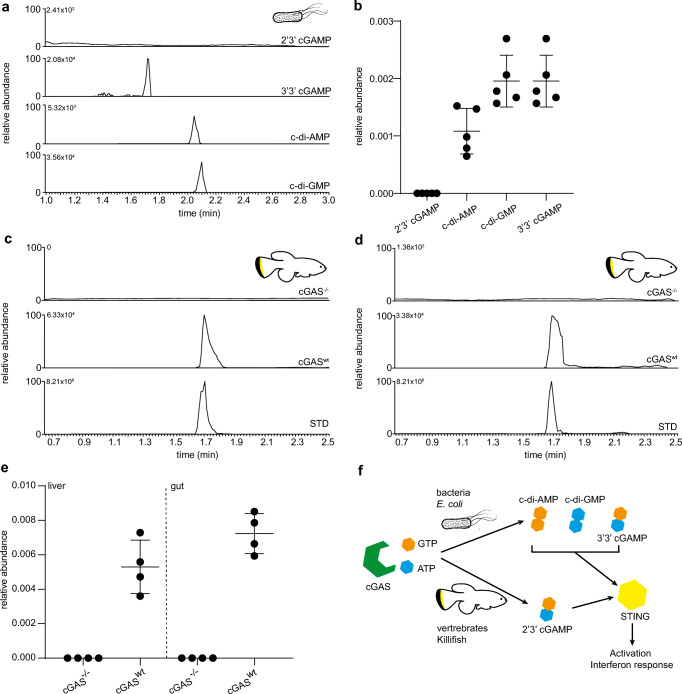
Method validation and quantification of CDNs in vivo. **a** Quantification of CDNs in *E. coli* using the optimized LC-MS/MS parameters. **b** Relative abundance of CDNs is calculated by dividing the CDN peak area with the internal standard peak area and further normalized to protein levels. Quantification of 2′3′ cGAMP in liver (**c**) and gut (**d**) of killifish by LC-MS/MS. The first chromatogram is obtained from the mutant with non-functional cGAS (cGAS^−/−^), the second chromatogram from the laboratory wild-type killifish GRZ-Ad (cGAS^wt^). The bottom chromatogram corresponds to the 2′3′ cGAMP standard. **e** Relative quantification of 2′3′ cGAMP in the killifish tissues. Relative quantification is calculated by dividing the CDN peak area with the internal standard peak area and further normalized to protein levels. **f** Representation of the cGAS/Sting pathways in bacteria and vertebrates. Bacteria synthesize only c-di AMP, c-di-GMP, and 3′3′ cGAMP, whereas vertebrates only synthesize 2′3′ cGAMP

In order to test the validity of the method, we used the optimized LC-MS/MS MRM parameters to quantify CDNs in biological samples. We first quantified endogenous 3′3′ cGAMP, c-di-AMP, and c-di-GMP from extracts of the bacteria *E. coli* (OP50 strain). The spectra clearly showed the presence of these three CDNs (Fig. 4a) within complex mixtures. The relative quantification of these species revealed higher levels of c-di-GMP and 3′,3′ cGAMP in comparison to the c-di-AMP. As expected, the 2′3′ cGAMP isomer was absent (Fig. 4b).

We then carried out the quantification of 2′3′ cGAMP in the killifish *Nothobranchius furzeri*. We examined compound levels in two tissues (liver and gut) of the laboratory strain GRZ-AD. Using CRISPR/Cas9 technology, we also generated a viable cGAS knock-out (cGAS^−/−^) mutation, which disrupted the open reading frame and served as a negative control lacking the enzyme-producing endogenous 2′3′ cGAMP. As expected, the 2′3′ cGAMP peak was completely absent in the cGAS^−/−^ knock-out line. In wild type, the MS spectra revealed the presence of a peak at the 1.71 min retention time similar to the standard peak of 2′3′ cGAMP (Fig. 4c, d). 2′3′ cGAMP levels appeared slightly higher in the gut compared to the liver (Fig. 4e), though this difference did not reach significance.

## Discussion

The present study illustrates a powerful new method to quantify cyclic dinucleotides in biological samples. Our method agrees well with previous work, which used similar chromatographic phases [21, 22]. In addition, by deploying the specific CE-breakdown properties of the four cyclic dinucleotides, we identified for the first time new diagnostic fragments of the four CDNs, namely ion at *m*/*z* 476.1 for 2′3′ cGAMP, ion at *m*/*z* 428.0 for 3′3′ cGAMP, ion at *m*/*z* 250.1 for c-di-AMP, and ion at *m*/*z* 248.1 for c-di-GMP. Importantly, the transition selection and CE parameter optimization for each compound increased the sensitivity of our method compared to previous works by 1.4–2.5-fold [22].

In our studies, we observed that the CDNs show different profiles in the low and high collision energy ranges. Harrison et al. and others used a similar approach to obtain energy-resolved fragmentation data from CID experiments of tripeptides [26–28]. We surmise that differences in the observed product ion intensity at higher collision energies reflect the different internal energy of the four molecules. Conceivably, the bond formed at 2′3′ or 3′5′ as well as the different hybridization properties of guanosine and adenosine moieties affect the energetic character and stability of the molecule. As shown by Green-Church and colleagues, the nucleobases adenine and guanine have slightly different proton affinities, and this property could affect the appearance of specific signals in the tandem mass spectra [29].

The fragmentation spectra of an ion are rich in information as it reflects the relative energy of the bonds present as well as the structure of both the precursor and product ions. Surprisingly, c-di-GMP did not produce as many fragments compared to other CDNs under our conditions. Conceivably, c-di-GMP might have slightly lower proton affinities, which could lead to the reduced formation of product ions. It is also possible that the two guanosine moieties increase the stability of c-di-GMP, rendering it less susceptible to CID compared to the other CDNs.

CDNs in metazoans function as endogenous second messengers that provoke multiple signaling cascades [30]. Though the enzymes for the synthesis of CDNs are evolutionarily conserved across species, the nucleotides used for the cyclization reaction and the cyclization bond differ somewhat between bacteria and vertebrates [2] (Fig. 4f).

Our quantification of CDNs in *E. coli* is in good agreement with previous work. It has been described previously that Gram-negative *E. coli* bacteria have higher levels of 3′3′ cGAMP and c-di-GMP compared to c-di-AMP. Karaolis et al. describe a role of c-di-GMP in metal-reducing activity and host colonization [31]. Low levels of c-di-GMP are associated with flagella-based motility, and high levels promote biofilm production [4]. While bacterial synthesis of CDNs often occurs constitutively under normal physiological conditions and is essential for various aspects of growth and metabolism, they can also be regulated as part of the bacterial antiviral response [1].

In vertebrates, these bacterial produced CDNs can serve as pathogen-associated molecular patterns, which are able to directly activate STING and trigger innate immune defense upon infection [32]. In addition, cGAS can detect the inappropriate presence of DNA in the cytosol, and produce 2′3′ cGAMP to activate STING, and downstream effector pathways of interferon response and immune defense. Sources of cytosolic DNA can be exogenous, such as during viral infection, or endogenous, such as mitochondrial DNA (mtDNA), micronuclei, and chromosomal fragments that arise under conditions of genome instability and aging [1, 33–35], leading to chronic inflammation and senescence [36]. The ability to accurately measure these CDNs should enable a clearer understanding of their dynamics in vivo in various contexts.

To evaluate the 2′3′ cGAMP concentration in vertebrates, we used the African turquoise killifish *Nothobranchius furzeri* as a model system. The inbred laboratory wild-type strain of this species, GRZ-AD, lives approximately 6 to 7 months, making it among the shortest-lived vertebrates kept in captivity [23, 37]. *Nothobranchius* is readily amenable to genetic manipulation, since transgenesis and crispr-mediated genome engineering are nearly as facile as other model organisms [38, 39]. We report here for the first time the generation of a cGAS^−/−^ knock-out line in the killifish, demonstrating conservation of its biochemical activity in vivo. Using our new method, we observed that 2′3′ cGAMP is absent from the liver and gut tissues of cGAS^−/−^ knock-out fish, providing clear genetic evidence that cGAS is the sole enzyme synthesizing 2′3′ cGAMP in this organism.

To date, 2′3′ cGAMP has been quantified mostly in in vitro cell culture models and only a handful of groups have used methods of detection from whole animal tissues [30]. These methods require more starting material, and have multiple extraction and enrichment steps that are quite labor intensive. By comparison, we developed a relatively simple and efficient method for the quantification of the four known natural CDNs from vertebrate tissues as well as bacterial cells [40], allowing a scale-down of sample preparation and greater sensitivity. Further, our procedure does not require heat treatment, which might affect the stability of CDNs [41]. With this method, future studies can illuminate CDN dynamics in various tissues and organisms, and thus help elucidate their physiological impact in organismal health.

## Conclusion

In the current study, we established a highly sensitive mass spectrometry–based method for the quantification of CDNs in vivo. Using product ion scans in combination with CE-breakage curves, we unraveled the mass spectrometric behavior of four biological relevant CDNs. This work can pave the way to further investigate the diverse energy states and stability of CDNs.

Our studies should greatly facilitate the measurement of CDN levels in different model organisms as well as in human tissues. In particular, because CDNs are key factors in the immune response, their accurate measurement may help reveal fundamental aspects of immune signaling and immunosenescence. Finally, in the future, it will be interesting to further characterize CDN functions and their physiological impact using the cGAS knock-out mutants in the killifish.

## Supplementary information

ESM 1 (DOCX 309 kb)
