# Supplementary material for: Mass spectrometric characterization of cyclic dinucleotides (CDNs) in vivo
Source: Anal Bioanal Chem. 2021 Sep 2;413(26):6457–68. doi: 10.1007/s00216-021-03628-6 (PMC8412381; doi:10.1007/s00216-021-03628-6)
Supplement: Supplementary file 1 — (DOCX 309 kb) [file 216_2021_3628_MOESM1_ESM.docx]

**Supplementary Figure 1**

**
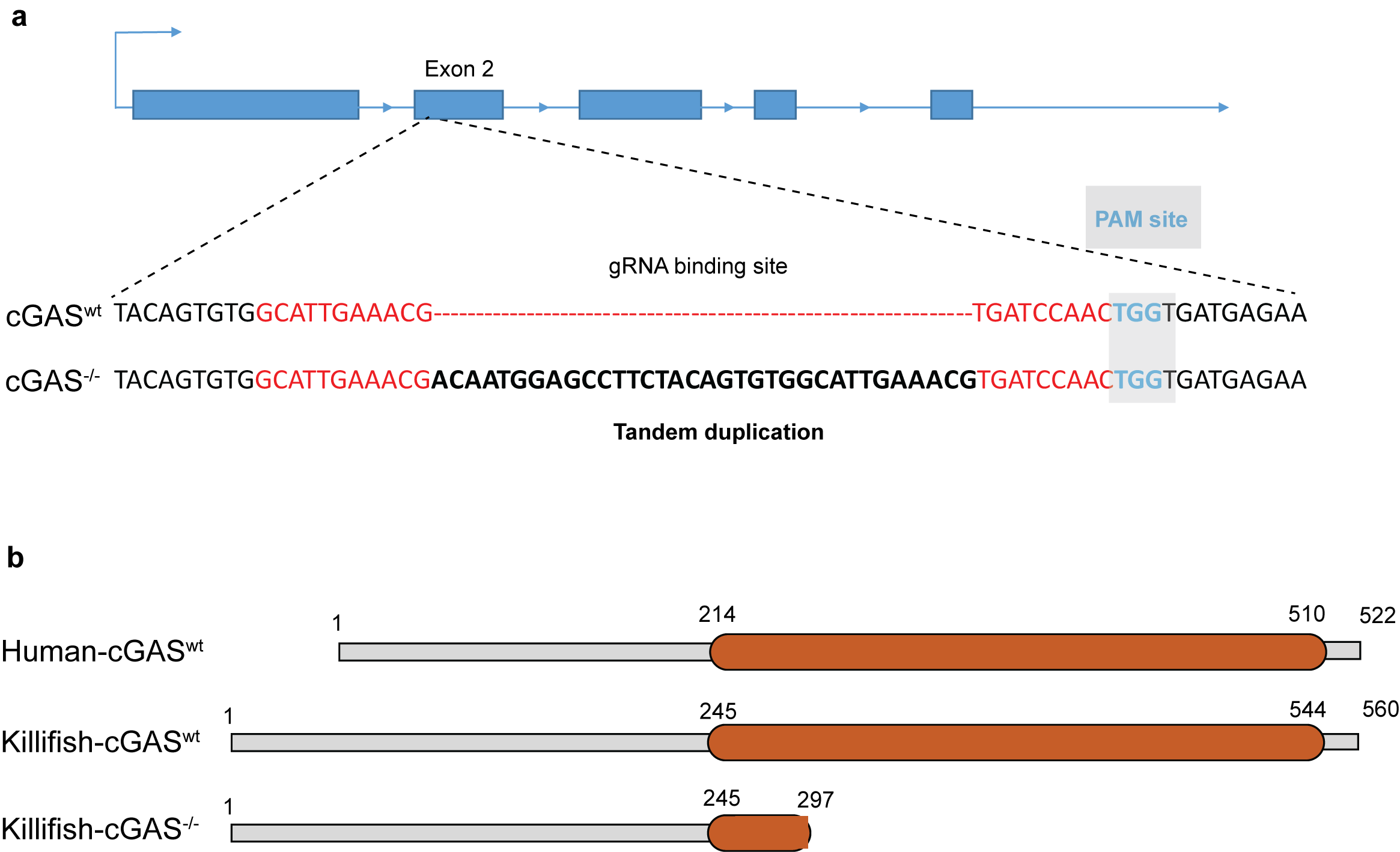
**

**Sup Fig. 1. Crispr-generated knockout of *N. furzeri* cGAS gene (a**), A tandem duplication of 34bp (shown in bold) generated by crispr-mediated cleavage in the cGAS gene leads to a frameshift, resulting in multiple stop codons and truncation of the protein.**(b),** Protein architecture of human cGAS and killifish cGAS. The mutated killifish cGAS is stopped prematurely, truncating the C-terminal NTAse core and MAB21 domains (shown in orange) required for enzymatic activity.

**Supplementary Figure 2**

**
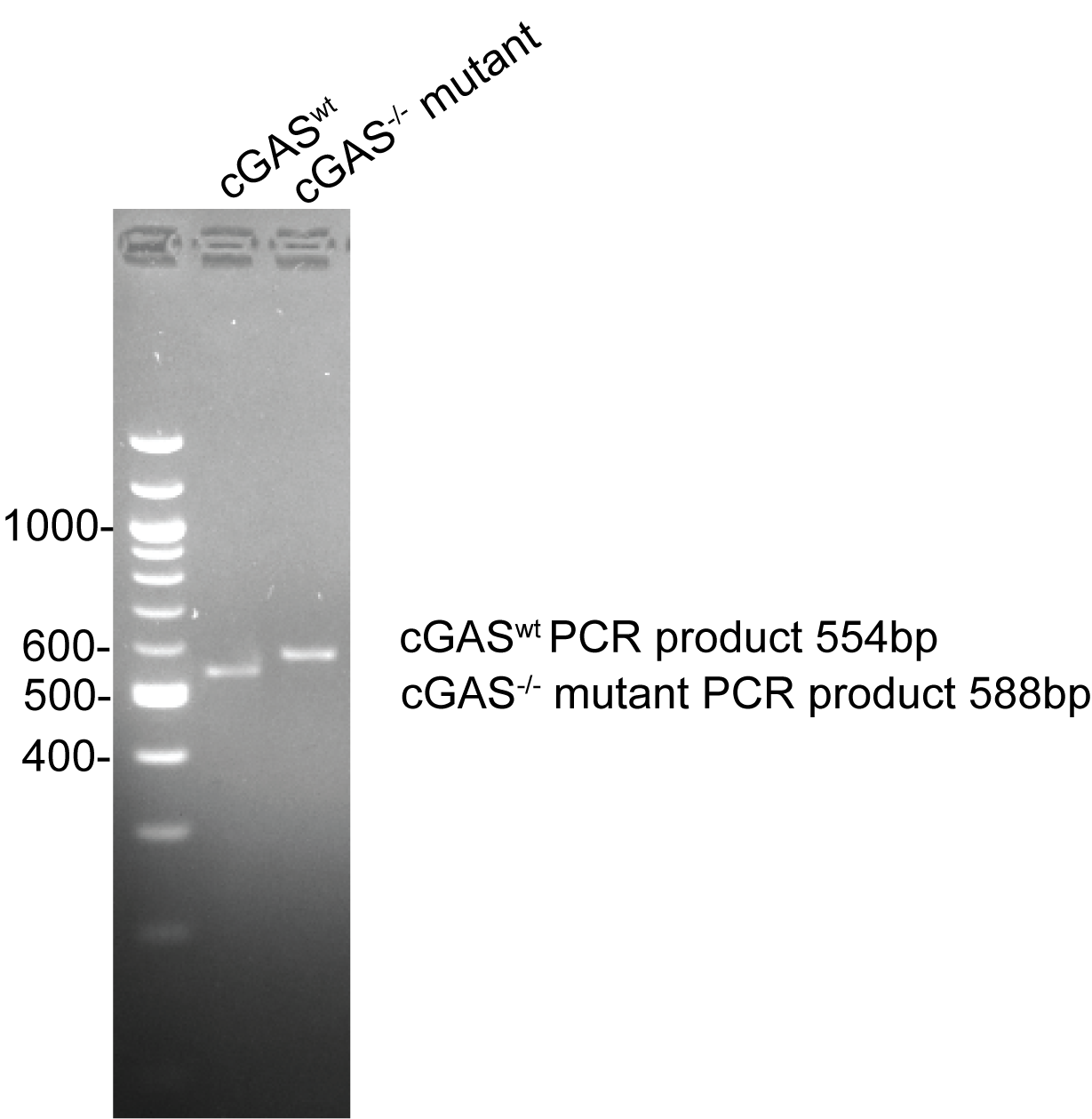
**

**Confirmation of cGAS^-/-^:** Genomic DNA from both cGAS^wt^ and cGAS^-/-^ was amplified with Forward primer: GTTAAGGAACCCCTTCGCACT and Reverse primer: TTGCCGTCATCTCCCATTCTG. PCR product was separated on 2.5% Agarose gel, 50V for 2 hr. The cGAS^wt^ DNA exhibits a PCR product at 554bp, whereas the cGAS^-/-^ DNA, which contains a 34 bp duplicated sequence, has a PCR product at 588bp.
